# Influenza Surveillance in the Central African Republic From 2015 to 2018 to Inform Vaccination and Treatment Strategies

**DOI:** 10.1111/irv.70221

**Published:** 2026-01-09

**Authors:** Giscard Francis Komoyo, Chantal J. Snoeck, Rod S. Daniels, Marie R. Belizaire, Judith M. Hübschen, Claude P. Muller, Pierre Somse, Yap Boum, Emmanuel Nakoune, John W. McCauley

**Affiliations:** ^1^ Laboratory for Arboviruses, Viral Hemorrhagic Fevers, Emerging Viruses and Zoonoses Institut Pasteur de Bangui Bangui Central African Republic; ^2^ Clinical and Applied Virology Group, Department of Infection and Immunity Luxembourg Institute of Health Esch‐sur‐Alzette Luxembourg; ^3^ The Worldwide Influenza Centre The Francis Crick Institute London UK; ^4^ School of Medicine University of Alcala Madrid Spain; ^5^ The World Health Organization Country Office Bangui Central African Republic; ^6^ Ministry of Health and Population Bangui Central African Republic; ^7^ The Institut Pasteur de Bangui Bangui Central African Republic

**Keywords:** antigenic analysis, antivirals, genetic diversity, influenza, phylogeny, seasonality, surveillance

## Abstract

**Background:**

Surveillance of influenza infections and virus characterisation are essential to guide prevention strategies. In the Central African Republic (CAR), data on influenza viruses are patchy and poorly documented.

**Objective:**

To study the clinical, seasonal, genetic and phenotypic characteristics of influenza viruses circulating in the CAR population.

**Materials and Methods:**

From January 2015 to December 2018, the presence of influenza A and B viruses in patients presenting with influenza‐like illness (ILI) symptoms or severe acute respiratory infections (SARI) was investigated by RT‐qPCR. Influenza genetic diversity was evaluated by phylogenetic analyses, and antigenic properties were investigated by haemagglutination inhibition assays, whereas reduced susceptibility to neuraminidase inhibitors was assessed through the presence of known genetic markers and neuraminidase assay. The relationship between patients' clinical characteristics and infection status was investigated using statistical analyses.

**Results:**

Over the surveillance period influenza viruses were detected in 9.7% of samples (*n* = 6134), with the highest intensity of circulation occurring in 2016 (15.8%), attributed mainly to A(H3N2). Periods of increased influenza transmission (June to October) generally coincided with rainy seasons; however, variations in terms of monthly distribution of cases between years were evident. Hospitalisation rates (SARI) were most frequent in infants (0–11 months, 37.9%) and young children (1–4 years, 24.8%), whereas influenza prevalences were highest in the 15–49 (12.0%) and ≥ 50 (15.2%) years old categories. A new A(H1N1)pdm09 6B.1 hemagglutinin subgroup characterised by amino acid substitutions S84N, S162N and I216T was detected in 2015, with associated antigenic drift, and subsequently, two of these viruses showed highly reduced inhibition by oseltamivir.

**Conclusion:**

This study showcases the value of local influenza sentinel networks to specifically inform vaccination strategies and highlights the need for improved strain characterisation in tropical regions.

## 1 Introduction

Seasonal influenza A and B viruses give rise to epidemics of respiratory infections worldwide and are major causes of hospitalisation and death among at‐risk individuals, including young children, the elderly, pregnant women, the immunocompromised and the chronically ill [1, 2]. The World Health Organisation (WHO) estimates between 290,000 and 650,000 deaths annually attributable to seasonal influenza [3, 4]. A retrospective analysis of seasonal influenza epidemics between 2010 and 2019 revealed that in 25 African countries, influenza viruses were responsible for 28,000–163,000 deaths per year [5]. In the Central African Republic (CAR), respiratory infections accounted for 16% of consultations and 15% of deaths in 2022 according to the WHO country office [6].

Influenza types A and B viruses have single‐stranded RNA genomes consisting of eight negative sense segments encoding at least 11 different proteins (https://ictv.global/). The main surface glycoproteins, hemagglutinin (HA/H) and neuraminidase (NA/N), play major roles in the infectious process and are the main targets of neutralising antibodies [7, 8, 9]. The HA is involved in binding to the target cell receptor and subsequent fusion of the virus lipid envelope with host cell membranes, whereas the NA releases the virion from the infected cell at the end of the replication cycle [10, 11]. The influenza B virus is divided into two lineages, Victoria and Yamagata [12], based on the genetic diversity of HA and does not cause pandemics due to lack of animal reservoir. Influenza A viruses are divided into 19 different HA and 11 NA subtypes [13, 14, 15], but the A(H1N1), A(H2N2) and A(H3N2) have been the predominant subtypes circulating in humans since the beginning of the 20th century. Influenza A viruses can lead to pandemics after antigenic shift resulting from the introduction of a new influenza strain of animal origin into the human population or reassortment between strains of human and animal origin. The latest pandemic was caused by A(H1N1)pdm09 that emerged from a reassortment between European and American swine strains probably in Mexico [16]. Since then, A(H1N1)pdm09 and A(H3N2) strains are co‐circulating together with influenza B strains and are responsible for seasonal influenza virus epidemics due to the emergence of variants that escape immunity conferred by vaccination or natural infection (antigenic drift). The genetic and antigenic heterogeneity is the consequence of rapid evolution through error‐prone replication and an accumulation of continuous mutations selected by escaping host immune pressure of neutralising antibodies on HA and NA [9, 17, 18, 19, 20, 21], as well as inter‐ and intra‐lineage reassortments. From a phylogenetic point of view, several virus clades have been identified within each subtype or lineage [22].

Despite the high burden of influenza epidemics in low‐ and middle‐income countries [23], the epidemiology of influenza viruses in tropical and subtropical climates is still far from completely understood. In particular, the seasonality patterns are much more diverse than in regions with temperate climates [24, 25], and a proper description at the national and subnational level is essential for optimising vaccination schedules. In addition, more efforts to characterise circulating influenza strains are needed to inform on vaccine formulation choice between northern or southern hemisphere (SH) compositions. In CAR, sentinel influenza surveillance has been established since 2008, and initial results from 2010 to 2015 showed a higher number of influenza cases during the rainy season (June to November), but still high heterogeneity between years was observed [26, 27]. Positivity rates in the studied population ranged from 5.7% in 1–2 years old to 11% in the 15–50 year age group [26]. However, no data on the characterisation or genetic diversity of influenza viruses circulating in the population are available. Our aim was thus to confirm the trends in incidence per age group and seasonality observed during the previous reporting periods, as well as to determine for the first time the genetic and antigenic properties of the strains circulating locally.

## 2 Materials and Methods

### 2.1 Sample and Data Collection

Samples were collected from patients visiting sentinel sites of the national influenza surveillance network in CAR from January 2015 to December 2018. The Centre National Hospitalier Universitaire Pédiatrique de Bangui (CPB) is the reference centre for severe acute respiratory infection (SARI). Both the CPB and the Centre de Santé Saint Joseph are located in the capital Bangui [28]. The other sentinel surveillance sites are located on two major routes serving the capital (Boali Health Center, Bossembélé Prefectural Hospital and Pissa Health Center). Sentinel surveillance sites other than CPB mainly screen patients presenting with influenza‐like illness (ILI) but also admit certain severe cases requiring hospitalisation, for referral the following day. Patients presenting with ILI or SARI were recruited, with ILI, ‘measured fever or history of fever equal to or greater than 38°C and cough, with symptom onset within the past 10 days’, or SARI, ‘measured fever or history of fever equal to or greater than 38°C and cough, with symptom onset within the past 10 days and requiring hospitalization’ case definitions of WHO. Following verbal consent, information on each patient was captured using a standardised survey form covering sociodemographic, clinical and epidemiologic data.

After collection, nasopharyngeal swabs were discharged in a tube containing universal transport medium (Copan, Italy) to preserve virus integrity. The tube was identified and stored at +4°C or sent immediately in a cold‐chain‐maintaining carrier box to the National Influenza Reference Center (CNR), located at the Institut Pasteur de Bangui, for testing. At the CNR, each sample was aliquoted into 1.5‐mL tubes; an aliquot of 140 μL was taken for nucleic acid extraction, another of 200 μL for virus isolation; and two tubes were stored at −80°C.

### 2.2 Nucleic Acid Extraction and RT‐qPCR

Nucleic acid extraction from nasopharyngeal samples using QIAamp viral RNA mini kits was performed according to the manufacturer's instructions (QIAGEN, Hilden, Ref: 52906, Germany). Real‐time RT‐qPCR was performed on RNA extracts using superscript III one‐step RT‐qPCR kits (Invitrogen, Ref: 11732088, USA) with the primers and probes kindly supplied by the Centers for Disease Control and Prevention (CDC), WHO Collaborating Centre, Atlanta, USA in the CDC Human Influenza Virus Real time RT‐qPCR Diagnostic Panel (CDC ref.: I‐007‐05, Atlanta, USA). Amplifications were performed on ABI prism 7500 fast thermocyclers as follows: a reverse transcription step of 50°C for 30 min and 95°C for 2 min, followed by 45 cycles at 95°C for 15 s and 55°C for 30 s. Samples testing positive for influenza A or B were then subjected to subtype‐specific RT‐qPCR to distinguish between influenza A(H1N1)pdm09 and A(H3N2) or influenza B/Yamagata and B/Victoria. The same chemistry and cycling conditions, with specific primer sets as supplied in the CDC kit, were used to identify influenza subtypes.

### 2.3 Virus Isolation and Subtyping

Influenza virus‐positive samples (200 μL) with Ct values ≤ 30 were inoculated onto Madin–Darby canine kidney (MDCK) cell monolayers in 12‐well plates under standard conditions [29]. Plates were then incubated at 37°C with 5% CO_2_ and observed daily for 9 days under a microscope to visualise cytopathogenic effect. Culture supernatants were harvested every 2 days, starting on Day 3, for haemagglutination and haemagglutination inhibition (HI) assays [29] or RT‐qPCR for subtyping. Supernatants were stored at −80°C for virus characterisation and sequencing at the Worldwide Influenza Centre (WIC).

### 2.4 Phenotypic Characterisation of Viruses

All clinical specimens were subjected to influenza virus sequence determination, and positive specimens, together with shipped virus isolates, were cultured on MDCK cells or the MDCK‐SIAT‐1 derivative [30] (engineered to over‐express α2,6‐sialic acid) for A(H3N2) viruses. Cell culture–propagated influenza viruses were examined antigenically by HI assay [29]. Assays were performed using four haemagglutinating units of influenza viruses with 0.75% guinea pig or turkey red blood cells (RBCs) and post‐infection ferret antisera raised against sets of reference viruses. The HI titre was defined as the reciprocal of the highest dilution of serum still providing 100% agglutination. Susceptibility to neuraminidase inhibitors was determined as described previously [31] with MUNANA purchased from biosynth.com (# M‐5507), oseltamivir carboxylate and zanamivir provided by their manufacturers (Roche and GlaxoSmithKline, respectively) using a Tecan Infinite Pro 200 fluorimeter and GraFit software (https://en.freedownloadmanager.org/Windows‐PC/GraFit.html).

### 2.5 Phylogenetic Analyses

Sequencing of influenza strains, as clinical specimens or virus isolates, was carried out at the WIC by Sanger technique and/or next‐generation sequencing [32]. The sequences from samples that yielded complete influenza HA and associated NA gene sequences were deposited in the EpiFlu database of the Global Initiative on Sharing All Influenza Data (GISAID: http://www.gisaid.org).

Phylogenetic trees of complete coding sequences of HA and NA were estimated using RaxML v8.2X (https://cme.h‐its.org/exelixis/software.html), followed by annotation with amino acid substitutions defining nodes using treesub (https://github.com/tamuri/treesub/blob/master/README.md). Trees were visualised using FigTree (http://tree.bio.ed.ac.uk/software/figtree/) and highlighted using Adobe Illustrator CC 2015.3. For HA, the signal peptide coding sequences were removed from the alignments to give mature HA amino acid numbering. Sequences from reference viruses, vaccine viruses and viruses from other continents were obtained from GISAID.

### 2.6 Data Analysis

Periods of increased influenza activities were determined using the proportion of positive cases per month. Periods of increased activity were defined as periods of at least 2 consecutive months with the monthly proportion of positive cases exceeding the annual median proportion of positive cases [24]. Statistical analyses were carried out using GraphPad v10.3.1 software on categorical variables using χ^2^ tests for comparing influenza prevalence between surveillance years. Odds ratios (OR) were calculated with χ^2^ test to compare prevalence according to sex, age group or case definition. *p*‐values < 0.05 were considered statistically significant.

## 3 Results

### 3.1 Participant Enrolment

From January 2015 to December 2018, a total of 6134 participants were enrolled. Similar numbers of patients were tested each year during the study period, ranging from 1363 in 2015 to 1631 in 2017. Over half of the participants (*n* = 3837, 62.6%) were children under 5 years old. The majority of participants (*n* = 4655, 75.9%) presented with symptoms corresponding to the ILI case definition, and 1479 (24.1%) were hospitalised with SARI, among which 136 (9.2%) were treated in intensive care units. The odds of hospitalisations (SARI) were significantly higher in infants (0–11 months: 647/1706, 37.9%) compared to all other age groups (OR ranging from 1.85 to 4.13; *p* < 0.001 for all) and in young children (1–4 years: 529/2131, 24.8%) compared to the older age groups (OR ranging from 2.03 to 2.23; *p* < 0.001 for all). There were 59 deaths among all patients recruited, including five patients presenting with ILI. Mortality rates were inversely proportional to age range (2.2% in 0–11 months, 0.8% in 1–4 years old, 0.4% in 5–14 years old).

### 3.2 Influenza Detection in Association With Age and Disease Severity

Influenza viruses were detected in 596/6134 (9.7%) samples tested. Similar detection rates were observed in males and females (Table 1). Participants in older age groups (15–49, 12.0% and ≥ 50 years, 15.2%) had higher chances of testing positive for influenza (Table 1), and this was attributed to a significant difference in A(H3N2) prevalence in these two groups. Young infants were also significantly less affected by A(H1N1)pmd01 (1.3%) compared to other age groups (2.8%–4.1%). No age‐related differences were observed for influenza B viruses (Figure S4). The majority of positive cases presented with ILI (451/587, 76.8%) and did not require hospitalisation, but influenza detection rates for ILI (451/4655, 9.7%) and SARI (136/1479, 9.2%) cases were similar (*p = 0.565;* Table 1). Among recorded deaths, nine (15.2%) patients had tested positive for influenza. All influenza‐related deaths occurred in children younger than 36 months, infected with influenza A(H3N2) (*n* = 2), A(H1N1)pdm09 (*n* = 4) and B type (*n* = 3, with 2/3 by B/Victoria‐lineage).

**TABLE 1:** Distribution of cases testing positive for seasonal influenza viruses in CAR 2015–2018, according to sex, age group, surveillance year and clinical presentation.

Demographic characteristics	No. of patients testing positive for any influenza/no. of patients (%)	OR (95% CI)	*p* ^a^	No. of patients testing positive for A(H1N1)pdm09 (% all influenza)	No. of patients testing positive for A(H3N2) (% all influenza)	No. of patients testing positive for influenza B (% all influenza)
Patients	596/6134 (9.7)			157 (26.3)	260 (43.6)	179 (30.0)
Sex						
M	292/2976 (9.8)	Ref	—	83 (28.4)	127 (43.5)	82 (28.1)
F	304/3158 (9.6)	0.98 (0.83–1.16)	0.806	74 (24.3)	133 (43.8)	97 (31.9)
Age (years)						
0–11 months	123/1706 (7.2)	Ref	—	23 (18.4)	59 (47.2)	43 (34.4)
1–4	191/2131 (9.0)	1.28 (01.02–1.62)	0.038	59 (30.1)	75 (38.3)	62 (31.6)
5–14	76/727 (10.5)	1.50 (1.11–2.01)	0.008	22 (28.6)	33 (42.9)	22 (28.6)
15–49	153/1280 (12.0)	1.73 (1.32–2.21)	< 0.001	41 (26.6)	73 (47.4)	40 (26.0)
≥ 50	44/290 (15.2)	2.26 (1.57–3.26)	< 0.001	12 (27.3)	20 (45.5)	12 (27.3)
Years						
2015	98/1363 (7.2)	—	—	40 (40.8)	41 (41.8)	17 (17.3)
2016	255/1618 (15.8)	—	< 0.001	37 (14.5)	148 (58.0)	70 (27.5)
2017	104/1631 (6.4)	—	0.377	5 (4.8)	9 (8.7)	90 (86.5)
2018	139/1522 (9.1)	—	0.058	75 (54.0)	62 (44.6)	2 (1.4)
Clinical cases						
ILI	451/4655 (9.7)	Ref		125 (27.3)	193 (42.1)	140 (30.6)
SARI	136/1479 (9.2)	0.94 (0.77–1.15)	0.565	32 (23.2)	67 (48.6)	39 (28.3)
Death	9/59 (15.4)			4 (44.4)	2 (22.2)	3 (33.3)

^a^ *p*‐values were calculated using the Chi2 test and values of less than 0.05 were considered significant.

### 3.3 Seasonal Circulation

**FIGURE 1:**
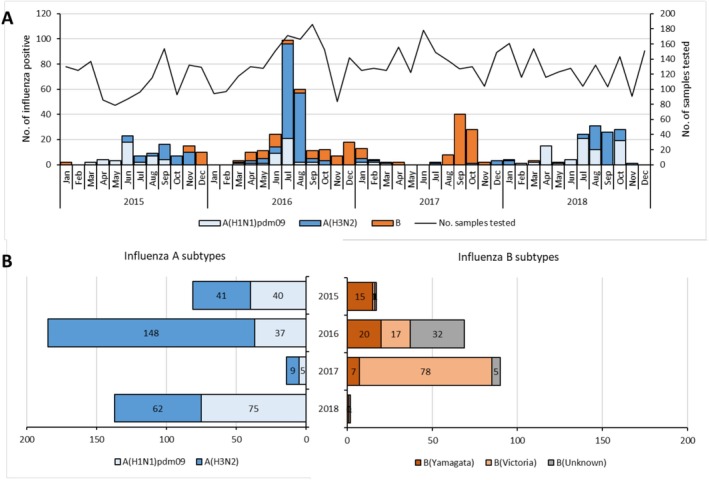
Seasonal influenza circulation in CAR by month between 2015 and 2018 (A) and according to (sub‐)types per year (B). The cumulative number of positive specimens are indicated by month or by year and colour‐coded by virus type/subtype. The total number of samples tested each month is indicated by the black line (secondary y‐axis on Panel A).

Overall, influenza viruses were detected during the observation period between January 2015 and December 2018 (Figure 1A). Across the 4 years of surveillance, Type A influenza viruses dominated over Type B viruses at a ratio of 2.3:1 (Table 1 and Figure 1B), but temporal differences were clearly evident. In detail, influenza viruses circulated almost uninterrupted but at low levels in 2015, with the absence of a clear peak in cases (Figure 1A,B). The season was marked by equivalent detections of A(H1N1)pdm09 and A(H3N2), and lower detection of B viruses, mainly B/Yamagata lineage, towards the end of the year. The 2016 season was the most intense of the 4 surveillance years, and the peak of cases was observed in July. The season was clearly dominated by A(H3N2) virus circulation, but co‐circulation of A(H1N1)pdm09 and B viruses was evident. Influenza B viruses tended to be more dominant towards the end of the year and circulated at an uninterrupted average level until April 2017. The 2017 season was clearly dominated by influenza B viruses, with almost exclusively B/Victoria lineage being detected. The peak of virus circulation happened in September. The 2018 season was more similar to the 2015 season. Influenza B strains were almost absent, whereas A(H1N1)pdm09 and A(H3N2) co‐circulated at equivalent levels.

In order to derive more general patterns of influenza activity in the country, periods of increased transmission were determined based on monthly proportions of positive cases (Table S1). As already visible in Figure 1A, periods of increased transmission during each year were variable in terms of number per year (mainly one period, but also a second in 2016–2017), in duration (from 3 to 5 months) and in timeliness. When aggregating the data over the 4 years of surveillance, the main periods of increased transmission took place between June and October, corresponding to the rainy seasons.

### 3.4 Characterisation of Influenza Viruses Detected in CAR

Influenza‐positive samples from 106 patients were shared with WIC (Table S2). Influenza virus and/or genetic information was recovered from 20 samples (recovery rate 20/106; 18.9%), for 15 A(H1N1)pdm09 and five A(H3N2) viruses. The poor influenza virus recovery rate at WIC, notably for Type B viruses, was probably associated with relatively high Ct values, issues relating to cold‐chain maintenance during transport/shipping and long‐term storage prior to arrival at WIC (Table S2).

#### 3.4.1 Antigenic Characterisation

HI assays were used to determine the antigenic similarity of CAR isolates to recommended vaccine viruses. A(H1N1)pdm09 viruses tested for this purpose were very well recognised by antisera raised against vaccine viruses A/California/7/2009 (7 strains from 2015 with ≤ 2‐fold reduced titre compared to homologous titre) and A/Michigan/45/2015 (6 strains from 2016 and 2018 with ≤ 2‐fold reduced titre compared to homologous titre) and by most of the other antisera (Tables S3 and S4). The one A(H3N2) virus isolated had no haemagglutinating activity, preventing HI testing. This issue was frequently encountered with A(H3N2) strains circulating in 2015–2018 [33].

#### 3.4.2 Genetic Characterisation

Phylogenetic analysis was used to determine genetic groupings of the CAR viruses and assess evolution compared to vaccine viruses.

#### 3.4.3 A(H1N1)pdm09 Viruses

**FIGURE 2:**
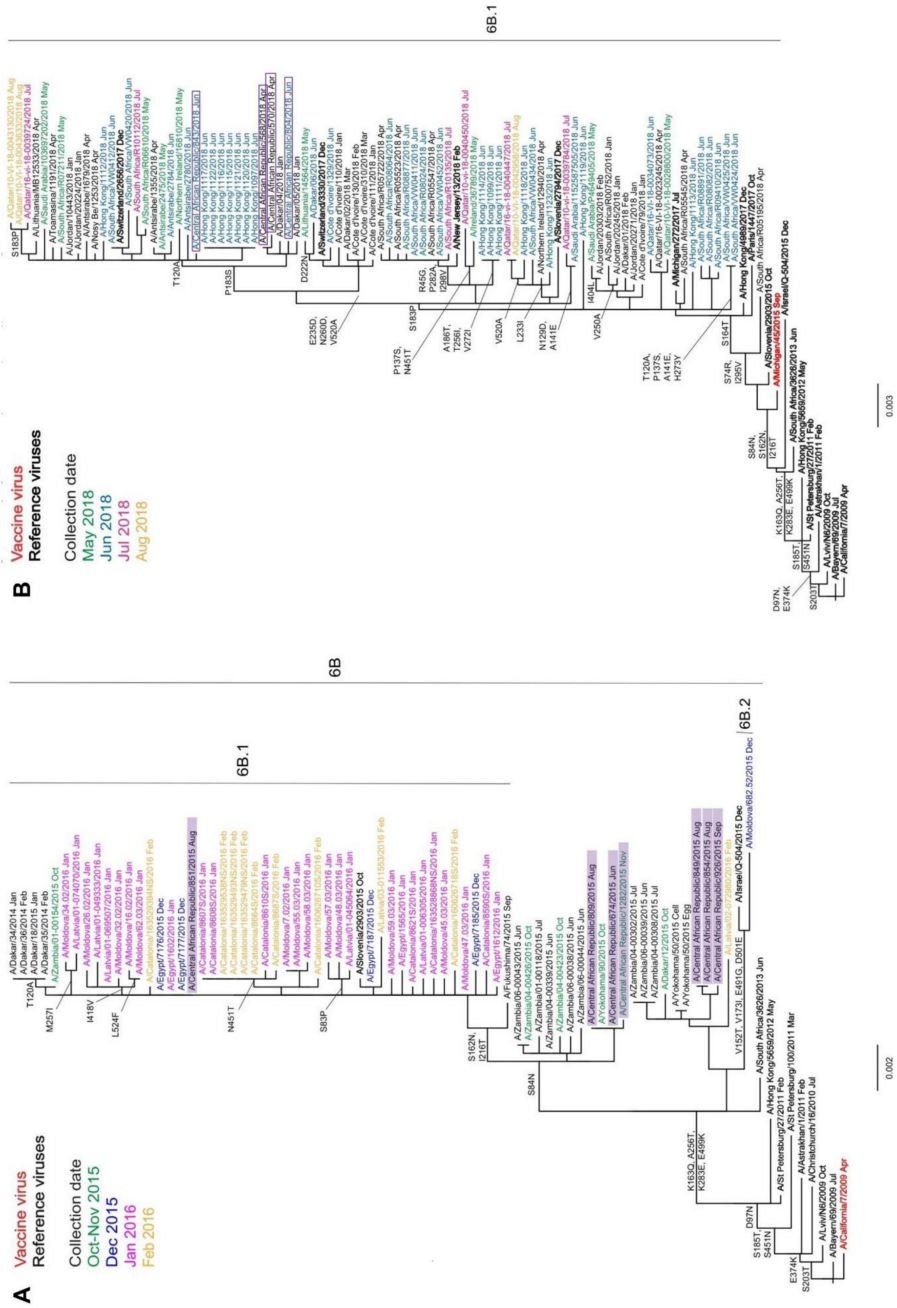
Phylogenetic analysis of A(H1N1)pdm09 HA genes for viruses detected in CAR. Both phylogenies are rooted on A/California/7/2009 and reference viruses (those to which post‐infection ferret antisera were raised) are shown in bold. Recommended vaccine strains are shown in red: (A) A/California/7/2009 for the southern hemisphere 2015 and viruses with collection dates from October 2015 to February 2016 are colour‐coded by month, with CAR viruses highlighted in purple; (B) A/Michigan/45/2015 and viruses with collection dates from May to August 2018 are colour‐coded by month, with CAR viruses framed in purple.

HA gene phylogeny of the seven CAR 2015 isolates showed they all belonged to genetic group 6B (Figure 2A). Three of the CAR group 6B viruses appeared to be precursors of the 6B.1 subgroup, carrying the S84N substitution in HA1 only (Figure 2A). A/CAR/851/2015, dating from August 2015, belonged to the then new genetic subgroup 6B.1 and represented an early member of this new subgroup first ascribed in October 2015. The NA gene sequences of the seven CAR 2015 viruses clustered similarly, falling into three subdivisions each defined by specific sets of amino acid substitutions (Figure S1A).

The four CAR 2018 viruses were also classified within the 6B.1 subgroup but further evolved, having amino acid substitutions S74R, S164T and I295V in HA1 (Figure 2B), with more subdivisions defined by additional specific substitutions, but with three main groupings along the trunk of the tree. Three CAR viruses belonged to a group defined by reversion of P183S in HA1, whereas the fourth CAR strain was defined by reversion of P183S and T120A substitution in HA1. Similar clustering was visible based on NA phylogenetic analyses (Figure S1B). All four CAR 2018 NA sequences were positioned off the T72I node and three of them contained additional substitutions of I108M and K260E, whereas the fourth carried an additional I365T substitution.

#### 3.4.4 A(H3N2) Viruses

**FIGURE 3:**
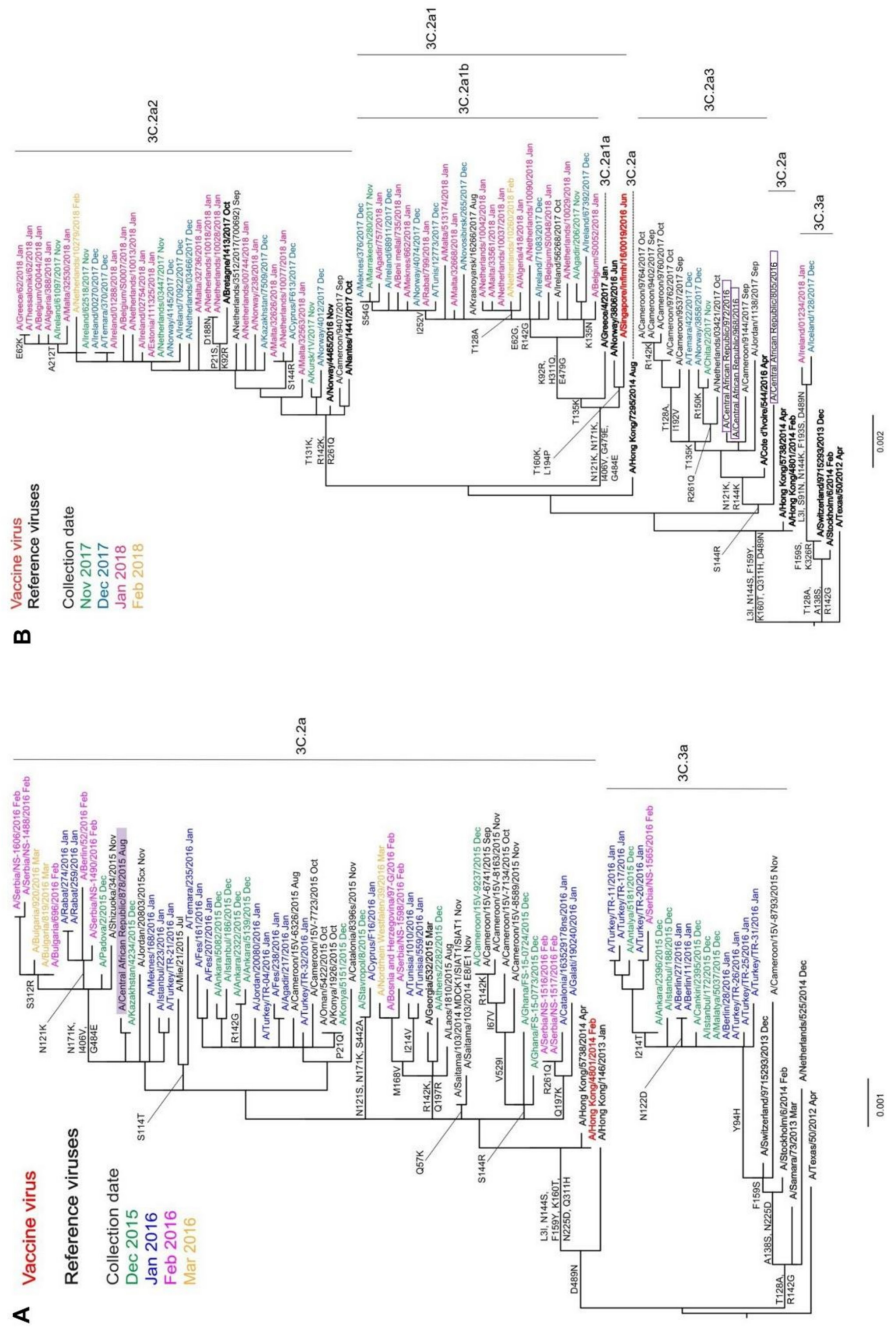
Phylogenetic analysis of A(H3N2) HA genes for viruses detected in CAR in 2015 (A) and 2016 (B). Reference viruses (those to which post‐infection ferret antisera were raised) are shown in bold; the trees are rooted on A/Texas/50/2012. The recommended vaccine virus for the 2016 and 2017 southern hemisphere A/Hong Kong/4801/2014 (Panel A) or 2018 southern hemisphere A/Singapore/INFIMH‐16‐0019/2016 (Panel B) are shown in red. CAR isolates are highlighted. Viruses with collection dates from December 2015 to March 2016 (A) or November 2017 to February 2018 (B) are colour‐coded by month.

The HA sequences of A(H3N2) viruses isolated worldwide in 2015 and 2016 fall into two distinct subgroups, 3C.2a and 3C.3a. A/Switzerland/9715293/2013, belonging to group 3C.3a, was introduced in 2015 for the SH vaccine composition, whereas the A/CAR/878/2015 virus belongs to group 3C.2a (Figure 3A), along with A/Hong Kong/4801/2014 first introduced as a vaccine strain for use in the 2016 SH vaccine composition, and was in a genetic group frequently associated with an inability to bind to RBCs.

Figure 3B shows a phylogeny made up largely of viruses from 2017 and 2018 and containing the three viruses collected in CAR in 2016. Significant genetic drift had occurred in subgroup 3C.2a, and a number of newly designated subgroups had emerged since 2015. Although A/CAR/805/2016 remained in subgroup 3C.2a, it had acquired an additional S144R substitution in HA1, and the other two viruses, A/CAR/968/2016 and A/CAR/972/2016, had also acquired HA1 N121K and R144K substitutions and fell into subgroup 3C.2a3. The sequences of the A(H3N2) viruses NA genes circulating in 2015–2016 clustered identically with that of A/CAR/878/2015 corresponding to HA subgroup 3C.2a (Figure S3A), whereas the two sequences from CAR viruses detected in 2016 (no sequence was available for A/CAR/805/2016) clustered together and corresponded to HA subgroup 3C.2a3 (Figure S3B).

#### 3.4.5 Amino Acid Sequence Changes

The HA and NA sequences of the CAR A(H1N1)pdm09 (*n* = 13) or A(H3N2) (HA: *n* = 4, NA: *n* = 3) strains were compared with those of the vaccine virus and representatives of specific A(H1N1)pdm09 genetic or A(H3N2) groups/subgroups, respectively, using FluSurver (https://flusurver.bii.a‐star.edu.sg/).

#### 3.4.6 A(H1N1)pdm09

Compared with A/Michigan/45/2015 egg‐propagated vaccine virus, HA amino acid sequence identity of CAR strains ranged from 99.1% to 99.8% for 2015 isolates, 99.8% for 2016 isolates and 98.9% to 99.3% for 2018 isolates, respectively, with all substitutions being in HA1. The seven 2015 CAR A(H1N1)pdm09 sequences contained up to five amino acid substitutions, all contained R223Q (223R being a substitution acquired on egg‐adaptation of A/Michigan/45/2015), six carried N162S (loss of a glycosylation site compared to A/Michigan/45/2015), five had T216I, three had N84S, and one had I298V. The two viruses from 2016 had an R223Q substitution only. The four 2018 viruses contained up to six substitutions: All carried S74R, S164T (modifying a glycosylation site), R223Q and I295V, whereas single HAs had additional substitutions of N38D or R45K and T299S.

Similarity to A/Michigan/45/2025 NA sequence ranged from 98.5% to 100% for 2015 isolates, from 99.4% to 99.6% for 2016 viruses and from 98.1% to 98.3% for 2018 isolates. Up to seven substitutions were observed in an individual sequence from 2015 isolates: Three contained V166I and V394I; two carried I13V, I117M, I264V, K270N, M314I, I365T and T438A; and one lacked the T438A substitution but had A75V. Both the 2016 viruses carried H275Y and I365T substitutions, and one had in addition G382E. The four 2018 sequences had six substitutions in common (T71I, G77R, V81A, I188T, D416N and N449D), whereas three also carried I108M and K260E, with one also having S285F, and a single sequence lacked I108M and K260E but carried V106I and I365T substitutions.

#### 3.4.7 A(H3N2)

Compared with the A/Hong Kong/4801/2014 mammalian cell‐propagated vaccine virus, the HA of the 2015 isolate showed 99.8% amino acid similarity with a single S262G substitution in HA1. Two of the 2016 viruses (A/CAR/968/2016 and A/CAR/972/2016) showed 99.6% homology with two amino acid substitutions in HA1 (N121K and S144K), whereas the third, A/CAR/805/2016, showed 99.1% homology with three substitutions in HA1 (H56Y, S144R and N216S) and two in HA2 (A43T and V200I). Compared with the A/Hong Kong/4801/2014 egg‐propagated vaccine virus, all four CAR virus HAs contained additional HA1 substitutions of S96N, K160T (a glycosylation site lost in the egg cultivar) and P194L (also the result of an egg cultivar‐specific substitution in the vaccine virus). The NA of the 2015 CAR isolate showed 98.5% amino acid similarity with that of both cultivars of the A/Hong Kong/4801/2014 vaccine virus, there being seven amino acid substitutions (I231V, S245N and S247T [creating a glycosylation site], T267K, I380V, T392I and P468H). A/CAR/968/2016 carried an additional three substitutions (97.9% homology, G93D, N161S and D339N), whereas A/CAR/972/2016 had a further two (97.4% homology, H36Y and S44P).

#### 3.4.8 Antiviral Susceptibility

CAR viruses were assessed for susceptibility to the NA inhibitors oseltamivir and zanamivir using genetic and phenotypic means (Table S5). All four A(H3N2) viruses showed normal inhibition (NI) by both antivirals. However, 2/15 A(H1N1)pdm09 viruses carried NA H275Y substitutions indicative of highly reduced inhibition (HRI) by oseltamivir. HRI status was confirmed in vitro for the available virus isolate. A/CAR/618/2016 was recovered from a specimen taken in June 2016 from a 15‐month‐old boy, and A/CAR/737/2016 was detected in a specimen taken in July 2016 from a 4‐year‐old boy. Both SARI patients had been hospitalised, but in different locations, and the outcome of their infections was not recorded. Importantly, neither patient had been treated with antivirals.

## 4 Discussion

Among the several potential factors proposed to influence seasonal influenza epidemics [34, 35], temperature, humidity and precipitation have been shown to play a major role in the survival and transmission of influenza viruses [36, 37]. In temperate zones, seasonal circulation of influenza is rather well defined and relatively homogeneous between years, with single rather short epidemics occurring in winter [38] when low temperatures and low humidity prevail [34]. In contrast, influenza epidemics in the tropics and subtropics are more difficult to predict being more variable in their number of occurrences, timing and duration and between countries even in the same region [39]. Increased influenza transmission tends to occur during the rainy season in Central Africa, as shown previously in Gabon and Cameroon [40, 41]. An overall similar trend was observed in the CAR, with periods of increased transmission taking place mostly on average between June and October during the 2015–2018 study period, similarly to 2012–2014 [26]. Therefore, timing vaccination in April, as currently recommended for the Western Africa influenza vaccination zone (that includes Cameroon, Nigeria, Togo, Benin, Côte d'Ivoire, Liberia, Sierra Leone, Guinea, Guinea‐Bissau, Senegal and CAR [42]), would confer immunity before the start of the primary period of influenza activity in CAR. However, circulation patterns in CAR were not homogeneous across years, and virus circulation was detected year‐round outside the periods of increased transmission. As influenza vaccine protection wanes over time due to declining immunity and/or antigenic drift, potential vaccination implementation in April in CAR would likely have diminished benefit for a non‐negligible number of infections taking place outside the periods of increased activity. Influenza circulation patterns also differed from those observed in Cameroon, the other country from the same influenza transmission zone with comparable levels of virus surveillance during that period (FluNet and [25, 41]). The influenza annual positivity rate in 2015 in CAR was not significantly different from 2017 or 2018, but virus circulation was moderate year‐round with a clear peak of transmission, whereas two epidemic waves took place in Cameroon in 2015. The 2016 CAR influenza season was more intense and clearly dominated by A(H3N2), whereas lower levels of virus circulation were noticed in Cameroon until the second half of 2017. The 2017 season was dominated by influenza B, notably B/Victoria lineage, in CAR but by A(H1N1)pdm09 in Cameroon, with peaks of circulation during overlapping periods between the two countries. Finally, the 2018 CAR season paralleled the 2015 season, whereas in Cameroon, one main period of increased transmission was noticed in October–November. Sentinel influenza surveillance in these two neighbouring countries located in the same transmission zone and with similar vaccination recommendations highlight once more the need for dedicated local surveillance to best inform and evaluate vaccination schedules.

In this study, we also performed genetic and antigenic characterisation of circulating influenza viruses in CAR based on complete HA and NA sequences, as well as phenotypic testing. Such investigations at national levels are essential to detect the emergence of viruses with different antigenic and antiviral susceptibility properties [32, 43]. After integration on a large scale, the generated results are critically important for recommendations on the composition of influenza vaccines for each hemisphere [44]. WHO currently recommends that influenza vaccination in CAR should use the SH vaccine formulation. Phylogenetic analyses of A(H1N1)pdm09 viruses detected in CAR from 2015 to 2018 showed them to match those in clades 6B and 6B.1 circulating in other parts of the world in the same periods, and they were antigenically similar to A/California/7/2009 (included in 2015 and 2016 SH and 2014–2015 till 2016–2017 NH vaccine formulations) and subsequently A/Michigan/45/2015 (included in 2017 and 2018 SH and 2017–2018 NH vaccine formulations) vaccine viruses. Interestingly, several A(H1N1)pdm09 strains from CAR in 2015 were precursors/early members of the 6B.1 subgroup, indicating the need for timely characterisation of influenza strains in the tropics to obtain early identification of emerging clades.

The predominance of subtype A(H3N2) in 2015 and 2016 coincided with the emergence of subclade 3C.2a. Although HI assays could not be used to assess the antigenicity of subclade 3C.2a viruses, more complex virus neutralisation assays showed such A(H3N2) viruses to be antigenically similar to the A/Switzerland/9715293/2013 (3C.3a) virus used in vaccines for the 2015 SH and the 2015–2016 NH seasons, but better recognised by antisera raised against A/Hong Kong/4801/2014 (3C.2a) used in vaccines for SH 2016 through to NH 2017–2018 [45]. CAR viruses showed HA and NA amino acid substitutions compared with the recommended vaccine strains, but the effects of these alterations on the escape mechanism of influenza viruses from patients' infection‐acquired immunity at country level are unknown. This is particularly so in countries like CAR that do not have a robust influenza vaccination programme. Generally, timely characterisation of influenza viruses circulating in CAR would assist in ensuring that use of SH vaccine formulations is most appropriate with the potential added advantage of identifying precursors of emerging virus clades.

Prevalences of influenza infections were significantly higher in participants ≥ 15 years in CAR. This contrasts with general observations that school‐aged children (5–14 years) are often considered as the drivers of influenza epidemics. Our observations may be confounded by the 2016 season and more frequent A(H3N2) infections in older participants. Indeed, the comparison of 32 influenza seasons in France [38] previously showed that the mean age of ILI cases was higher in epidemics dominated by A(H3N2), despite school‐age children being most affected by influenza overall when data for all (sub‐)types were aggregated. Similarly, a higher median age of infection with A(H3N2) compared to A(H1N1) or B viruses was also determined in an Australian cohort [46].

In this study, the majority of influenza viruses characterised in CAR during 2015–2018 showed sensitivity to neuraminidase inhibitors oseltamivir and zanamivir, except two strains from 2016. As these antivirals are not yet widely available in Central Africa, the detection of A(H1N1)pdm09 viruses resistant to oseltamivir in two children in CAR is notable. None of the 2018 strains were resistant, suggesting that they were not maintained locally. Resistance to NA inhibitors was not reported prior to their use [47]. However, reports described the emergence in 2008 of oseltamivir resistance of the former seasonal (pre‐2009) A(H1N1) viruses from patients not treated with antivirals [48, 49]. Examples of reduced sensitivity to oseltamivir have also been reported for A(H1N1)pdm09 viruses in localised clusters elsewhere [50, 51, 52]. Overall, this suggests that local independent emergence of resistance that was not maintained in CAR, although we acknowledge that the representativeness of the strain characterisation in our study is limited, hampering a definitive conclusion.

Our study has some limitations that affect the quality of our results. Patients often come to a hospital consultation after failure of any self‐treatments. A significant proportion of influenza viruses were detected in patients during their disease recovery phase, complicating virus sequencing and/or isolation. In addition, the sentinel surveillance system is located exclusively in or near the capital, and more regional variations in virus circulation are not captured. Hence, future developments should aim at improving the geographic coverage despite technical difficulties for sample preservation and transport from more remote areas.

## 5 Conclusion

The sentinel influenza network in CAR revealed year‐round circulation of influenza viruses, with heterogeneous periods of increased transmission regarding the intensity, peak and duration. Comparing with current vaccination recommendations, this study highlights that seasonal vaccination starting in April would adequately precede the main epidemics but would unfortunately lead to sub‐optimal immunisation for a not negligible number of infections occurring outside those periods. This study is a first contribution to the genetic and antigenic characterisation of influenza viruses in CAR. It illustrates the early emergence of a new antigenically diverged A(H1N1)pdm09 6B.1 genetic subgroup, highlighting the need to improve virus characterisation in the tropics for timely vaccination formulation evaluation. In the meantime, the data generated already enable health authorities to regularly sensitise the community and healthcare personnel in April–May, ahead of the main epidemic period, on non‐pharmaceutical interventions to prevent virus spread.

## Author Contributions

Conceptualisation: Giscard F. Komoyo, Chantal J. Snoeck, John W. McCauley, Yap Boum II and Emmanuel Nakoune. Data curation: Giscard F. Komoyo, Rod S. Daniels, Chantal J. Snoeck, Emmanuel Nakoune and Marie R. Belizaire. Formal Analysis: Giscard F. Komoyo, Chantal J. Snoeck, Rod S. Daniels and Marie R. Belizaire.

Funding acquisition: Emmanuel Nakoune, Pierre Somse and Judith M. Hübschen. Investigation: Giscard F. Komoyo. Methodology: Rod S. Daniels, Yap Boum II and Emmanuel Nakoune. Project administration: John McCauley, Yap Boum II and Emmanuel Nakoune. Resources: Giscard F. Komoyo, Rod S. Daniels, Judith M. Hübschen, John W. McCauley and Emmanuel Nakoune. Supervision: Chantal J. Snoeck, Judith M. Hübschen, Claude P. Muller, John McCauley, Rod S. Daniels, Yap Boum II and Emmanuel Nakoune. Validation: John McCauley, Yap Boum II and Emmanuel Nakoune. Visualization: Giscard F. Komoyo, Chantal J. Snoeck and Rod S. Daniels. Writing – original draft: Giscard F. Komoyo and Chantal J. Snoeck. Writing – review and editing: Giscard F. Komoyo, Chantal J. Snoeck, Claude P. Muller, Judith M. Hübschen, John W. McCauley, Rod S. Daniels, Yap Boum II and Emmanuel Nakoune.

## Funding

This work was supported by IRR, the US Department of Health and the CDC for funding the reagents and the Ministry of Foreign and European Affairs, Defence, Development Cooperation and Foreign Trade of Luxembourg under the grant ‘Microbiology for Development‐V’.

## Ethics Statement

The surveillance programme carried out in CAR was approved by the ethical committee of the Ministry of Health, Central African Republic (Decree 0277/MSPP/CAB/DGSPP/DMPM/SMEE of 5 August 2002). Participants were only included in the study after obtaining verbal consent and for children from their parents or legal guardians. Data were pseudo‐anonymised, in strict compliance with patient privacy rights.

## Conflicts of Interest

The authors declare no conflicts of interest.

## Supporting information


**Figure S1:** Phylogenetic analysis of A(H1N1)pdm09 NA genes for viruses detected in CAR in 2015. Reference viruses (those to which post‐infection ferret antisera were raised) are shown in bold, and the tree is rooted on the then current vaccine virus, A/California/7/2009, shown in red. Viruses with collection dates from October 2015 to February 2016 are colour‐coded and CAR isolates are highlighted.
**Figure S2:** Phylogenetic analysis of A(H1N1)pdm09 NA genes for viruses detected in CAR in 2018. Reference viruses (those to which post‐infection ferret antisera were raised) are shown in bold, the tree is rooted on A/California/7/2009, and the then current vaccine virus, A/Michigan/45/2015 (2018–2019 northern hemisphere influenza season), is shown in red. Viruses with collection dates from May to August 2018 are colour‐coded, and CAR isolates are framed in purple.
**Figure S3:** Phylogenetic analysis of A(H3N2) NA genes for viruses detected in CAR in 2015–2016. Reference viruses (those to which post‐infection ferret antisera were raised) are shown in bold, the trees are rooted on A/Texas/50/2012, and the then current vaccine viruses are shown in red. CAR isolates are highlighted. (A) CAR virus collected in 2015; those viruses with collection dates from December 2015 to March 2016 are colour‐coded. (B) CAR viruses collected in 2016; those viruses with collection dates from November 2017 to February 2018 are colour‐coded.
**Figure S4:** Prevalence of A(H1N1)pdm09, A(H3N2) and B/Victoria, B/Yamagata and untyped B infections per age group. A(H1N1)pdm09 prevalence was significantly different according to age groups (*p* = 0.003). A(H1N1)pdm09 prevalence in 0–11 months infants was significantly lower than in the four other age groups (*p* = 0.005 to *p* < 0.001). A(H3N2) prevalence was significantly different according to age groups (*p* = 0.002). A(H3N2) prevalence in 0–11 months infants was not significantly different than in 1–4 and 5–14 age groups but was significantly different than in 15–49 and ≥ 50 years old group. No significant difference between age groups were observed for B/Yamagata (*p* = 0.237) or B/Victoria (*p* = 0.139) infections were observed.

**Table S1:** CAR samples collected in 2015–2018 and received at WIC.
**Table S2:**: Antigenic characterisation of 2015 CAR A(H1N1)pdm09 isolates.
**Table S3:** Antigenic characterisation of post‐2015 CAR A(H1N1)pdm09 isolates.
**Table S4:**: Susceptibility of CAR Type A influenza viruses to neuraminidase inhibitors.

## Data Availability

The sequences generated in this study have been deposited in the Global Initiative on Sharing All Influenza Data (GISAID) EpiFlu database, available on the website (http://www.gisaid.org) under the following accession numbers: EPI_ISL_219614, EPI_ISL_219615, EPI_ISL_219616, EPI_ISL_219617, EPI_ISL_219618, EPI_ISL_219619, EPI_ISL_219620, EPI_ISL_308716, EPI_ISL_308717, EPI_ISL_308718, EPI_ISL_308404, EPI_ISL_311886, EPI_ISL_330798, EPI_ISL_330802, EPI_ISL_330807 and EPI_ISL_218939. Also, the information reported in this article comes from a database of the Ministry of Health of the Central African Republic used by the Institute Pasteur of Bangui within the framework of a partnership between these two institutions. The data are not publicly available because they contain information deemed to be confidential for the identity of the included subjects.
