# Supplementary material for: Influenza Surveillance in the Central African Republic From 2015 to 2018 to Inform Vaccination and Treatment Strategies
Source: Influenza Other Respir Viruses. 2026 Jan 9;20(1):e70221. doi: 10.1111/irv.70221 (PMC12789657; doi:10.1111/irv.70221)
Supplement: Supplementary file 1 — Figure S1: Phylogenetic analysis of A(H1N1)pdm09 NA genes for viruses detected in CAR in 2015. Reference viruses (those to which post‐infection ferret antisera were raised) are shown in bold, and the tree is rooted on the then current vaccine virus, A/California/7/2009, shown in red. Viruses with collection dates from October 2015 to February 2016 are colour‐coded and CAR isolates are highlighted. Figure S2: Phylogenetic analysis of A(H1N1)pdm09 NA genes for viruses detected in CAR in 2018. Reference viruses (those to which post‐infection ferret antisera were raised) are shown in bold, the tree is rooted on A/California/7/2009, and the then current vaccine virus, A/Michigan/45/2015 (2018–2019 northern hemisphere influenza season), is shown in red. Viruses with collection dates from May to August 2018 are colour‐coded, and CAR isolates are framed in purple. Figure S3: Phylogenetic analysis of A(H3N2) NA genes for viruses detected in CAR in 2015–2016. Reference viruses (those to which post‐infection ferret antisera were raised) are shown in bold, the trees are rooted on A/Texas/50/2012, and the then current vaccine viruses are shown in red. CAR isolates are highlighted. (A) CAR virus collected in 2015; those viruses with collection dates from December 2015 to March 2016 are colour‐coded. (B) CAR viruses collected in 2016; those viruses with collection dates from November 2017 to February 2018 are colour‐coded. Figure S4: Prevalence of A(H1N1)pdm09, A(H3N2) and B/Victoria, B/Yamagata and untyped B infections per age group. A(H1N1)pdm09 prevalence was significantly different according to age groups (p = 0.003). A(H1N1)pdm09 prevalence in 0–11 months infants was significantly lower than in the four other age groups (p = 0.005 to p < 0.001). A(H3N2) prevalence was significantly different according to age groups (p = 0.002). A(H3N2) prevalence in 0–11 months infants was not significantly different than in 1–4 and 5–14 age groups but was significantly different than in 15–4 [file IRV-20-e70221-s001.docx]

Supplementary material


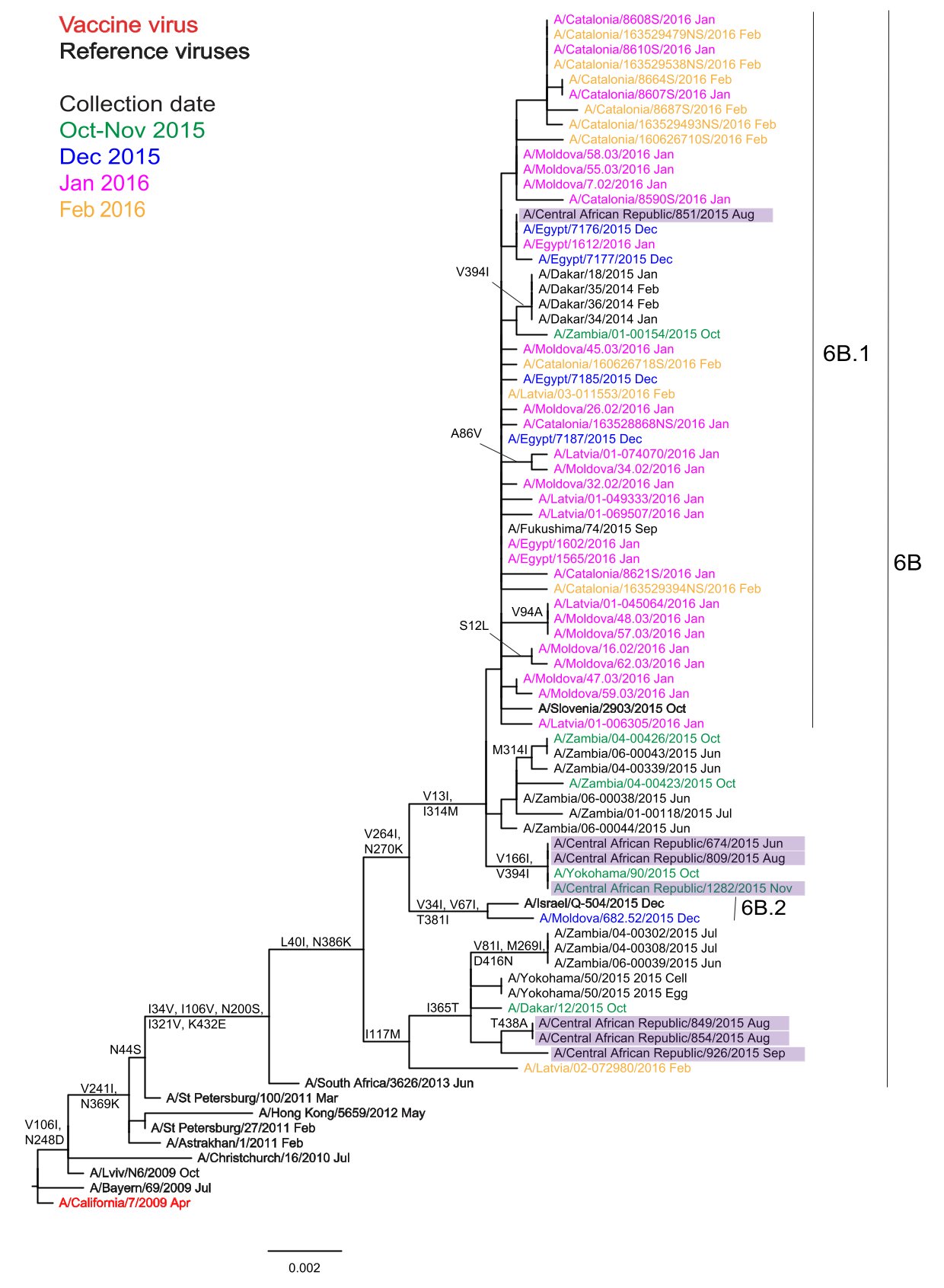


Figure S1: Phylogenetic analysis of A(H1N1)pdm09 NA genes for viruses detected in CAR in 2015.

Reference viruses (those to which post-infection ferret antisera were raised) are shown in bold and the tree is rooted on the then current vaccine virus, A/California/7/2009, shown in red. Viruses with collection dates from October 2015 to February 2016 are colour-coded and CAR isolates are highlighted.


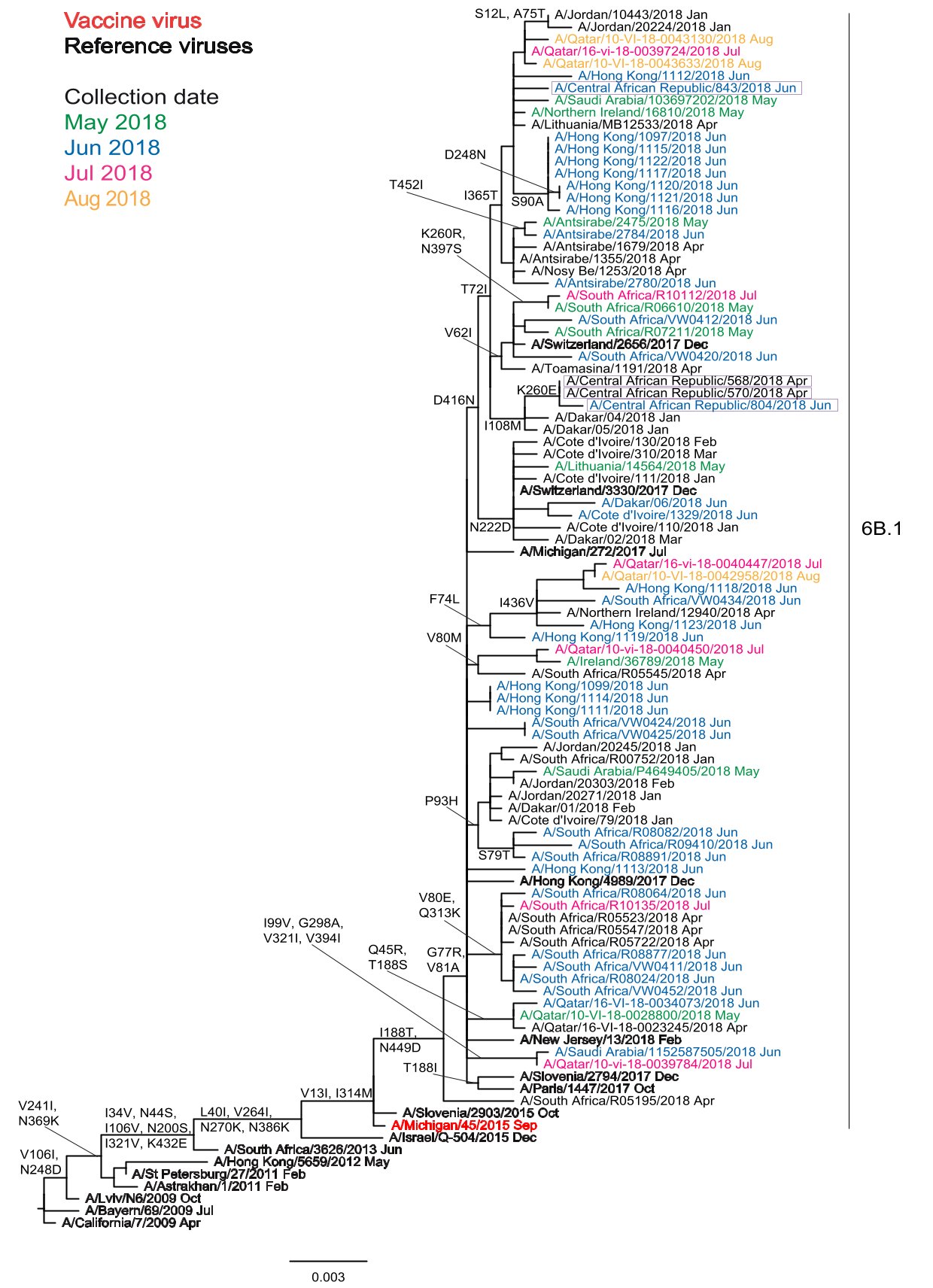


**Figure S2**: Phylogenetic analysis of A(H1N1)pdm09 NA genes for viruses detected in CAR in 2018.

Reference viruses (those to which post-infection ferret antisera were raised) are shown in bold, the tree is rooted on A/California/7/2009 and the then current vaccine virus, A/Michigan/45/2015 (2018-2019 northern hemisphere influenza season), is shown in red. Viruses with collection dates from May to August 2018 are colour-coded and CAR isolates are framed in purple.

| 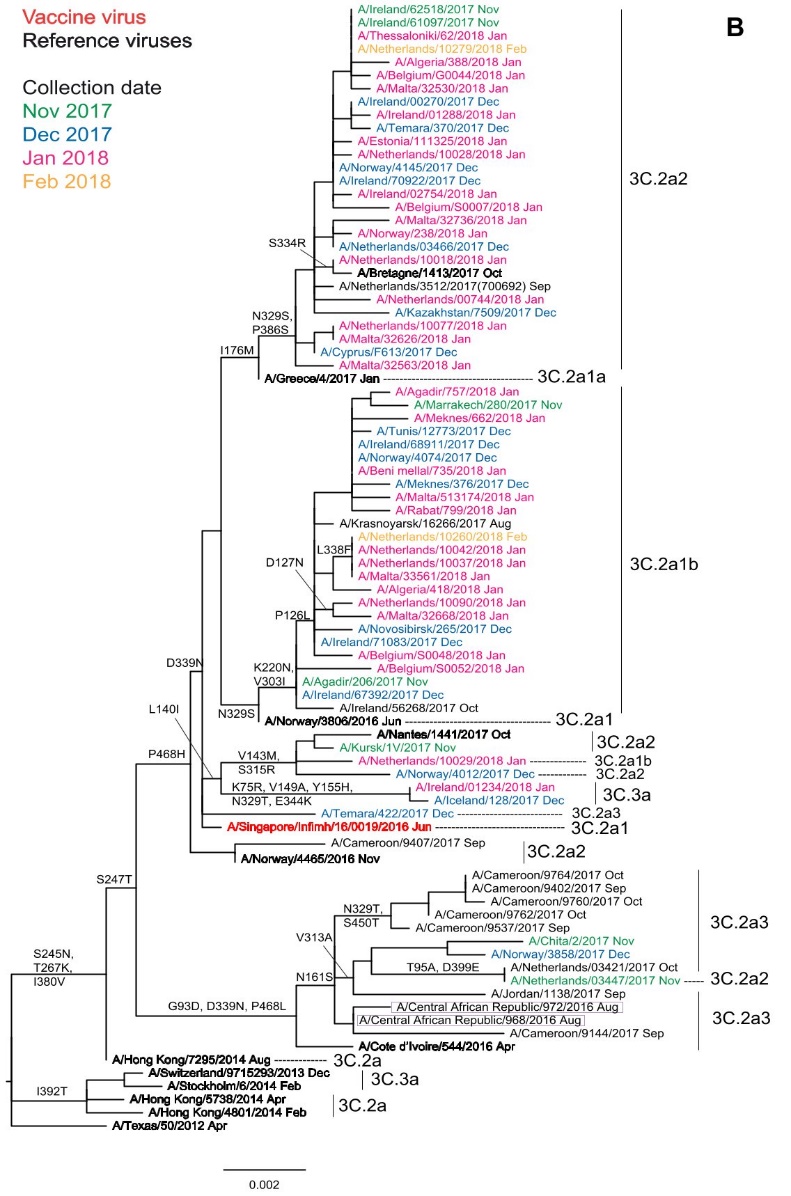 | **Figure S3**: Phylogenetic analysis of A(H3N2) NA genes for viruses detected in CAR in 2015-2016.  Reference viruses (those to which post-infection ferret antisera were raised) are shown in bold, the trees are rooted on A/Texas/50/2012 and the then current vaccine viruses are shown in red. CAR isolates are highlighted. A: CAR virus collected in 2015, those viruses with collection dates from December 2015 to March 2016 are colour-coded. B: CAR viruses collected in 2016, those viruses with collection dates from November 2017 to February 2018 are colour-coded. |
| --- | --- |
| 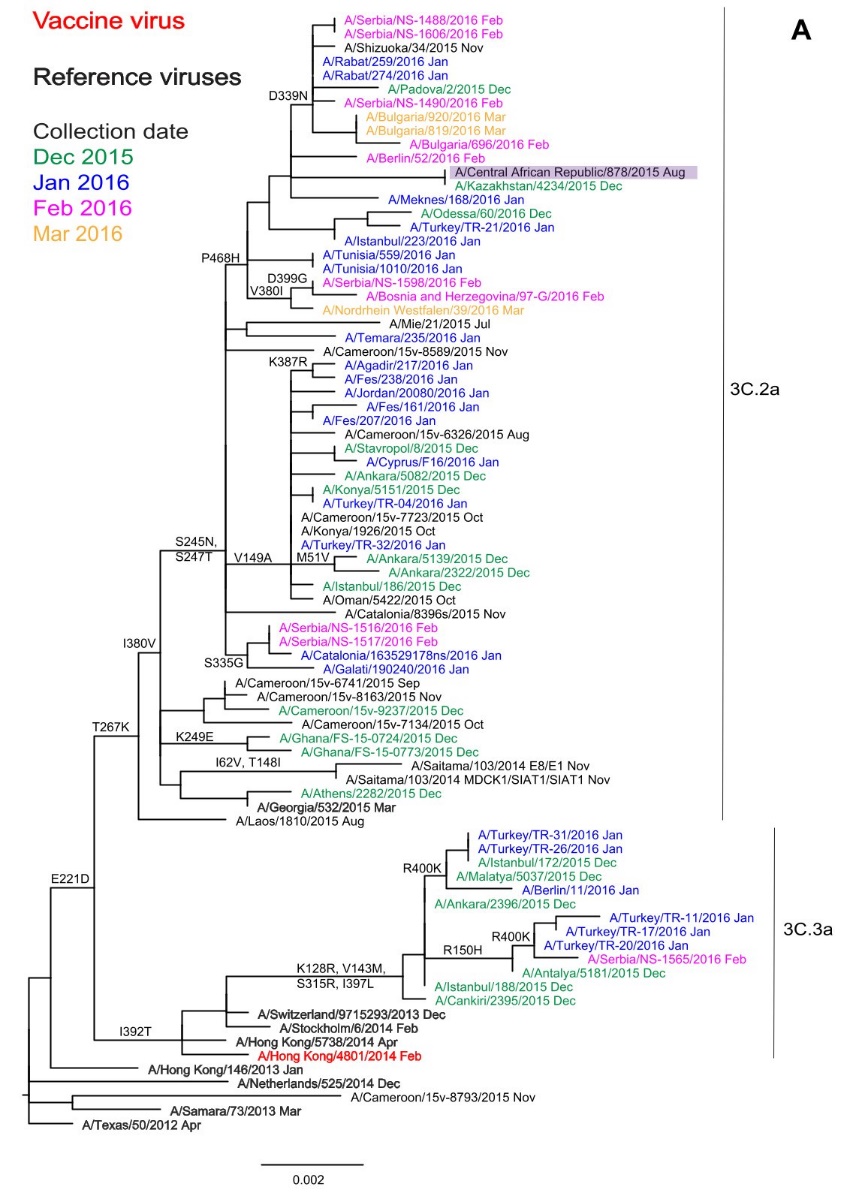 |  |


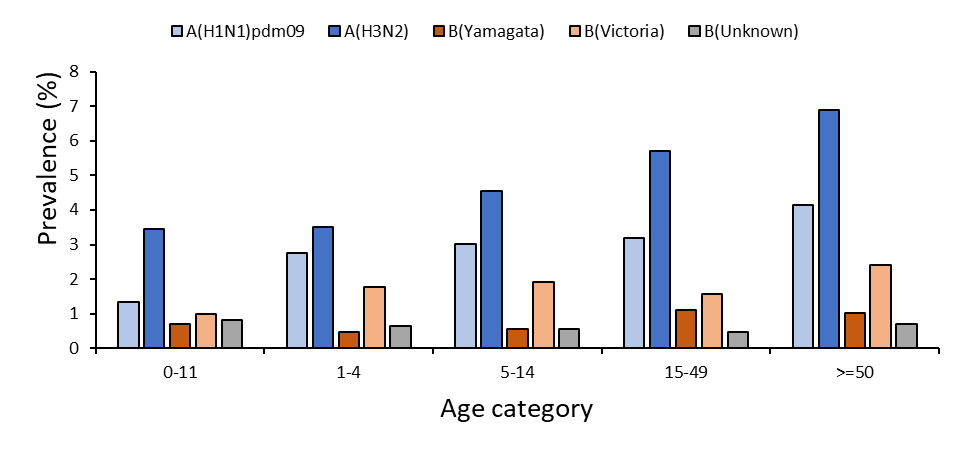


p=0.002

p=0.005

p<0.001

p=0.002

p<0.001

p=0.003

**Figure S4**. Prevalence of A(H1N1)pdm09, A(H3N2), and B/Victoria, B/Yamagata and untyped B infections per age group.

A(H1N1)pdm09 prevalence was significantly different according to age groups (p=0.003). A(H1N1)pdm09 prevalence in 0-11 months infants was significantly lower than in the four other age groups (p=0.005 to p<0.001). A(H3N2) prevalence was significantly different according to age groups (p=0.002). A(H3N2) prevalence in 0-11 months infants was not significantly different than in 1-4 and 5-14 age groups, but was significantly different than in 15-49 and ≥50 years old group. No significant difference between age groups were observed for B/Yamagata (p=0.237) or B/Victoria (p=0.139) infections were observed.
