# Supplementary material for: Influenza Surveillance in the Central African Republic From 2015 to 2018 to Inform Vaccination and Treatment Strategies
Source: Influenza Other Respir Viruses. 2026 Jan 9;20(1):e70221. doi: 10.1111/irv.70221 (PMC12789657; doi:10.1111/irv.70221)
Supplement: Supplementary file 2 — Table S1: CAR samples collected in 2015–2018 and received at WIC. Table S2:: Antigenic characterisation of 2015 CAR A(H1N1)pdm09 isolates. Table S3: Antigenic characterisation of post‐2015 CAR A(H1N1)pdm09 isolates. Table S4:: Susceptibility of CAR Type A influenza viruses to neuraminidase inhibitors. [file IRV-20-e70221-s002.docx]

Supplementary material

**Table S1**: CAR samples collected in 2015-2018 and received at WIC.

| **Collection Year** | **A(H1N1)pdm09** | | | | **A(H3N2)** | | | | **Influenza B** | | | |
| --- | --- | --- | --- | --- | --- | --- | --- | --- | --- | --- | --- | --- |
|  | **No received^$^** | **Success^!^** | **Average Delay*** | **Average Ct** | **No received^$^** | **Success^!^** | **Average Delay*** | **Average Ct** | **No received^$^** | **Success^!^** | **Average Delay*** | **Average Ct** |
| 2015 | 9(9V) | 7 | 176 | 30.3 | 8(8V) | 1 | 171 | 30.6 | 0 | - | - | - |
| 2016 | 12(5V,7cs) | 2 | 599 | 24.4 | 20(5V,15cs) | 4 | 562 | 26.6 | 1(1cs) | 0 | 555 | 27.3 |
| 2017 | 0 | - | - | - | 4(3V,1cs) | 1 | 90 | 24.6 | 28(28V) | 0 | 148 | 22.40 |
| 2018 | 21(3cs,18B) | 6 | 132 | 26.4 | 2(2V) | 0 | 234 | 28.5 | 1(1B) | 0 | 243 | 36.0 |
| **Total** | 42 | 15 |  |  | 34 | 5 |  |  | 30 | 0 |  |  |
|  |  |  |  |  |  |  |  |  |  |  |  |  |
| ^$^ Samples received as virus (V), clinical specimen (cs) or both virus and clinical specimen (B). | | | | | | | | | | | | |
| ^!^ Success is defined as virus being recovered or genetic information being generated from a clinical specimen. | | | | | | | | | | | | |
| * Number of days from time of specimen collection to receipt at WIC (average). | | | | | | | | | | | | |

**Table S2**: Antigenic characterization of 2015 CAR A(H1N1)pdm09 isolates.

|  |  |  |  | **Hemagglutination Inhibition Titre** | | | | | | | | | | |
| --- | --- | --- | --- | --- | --- | --- | --- | --- | --- | --- | --- | --- | --- | --- |
|  |  |  |  | **Post-infection ferret antisera** | | | | | | | | | | |
| **Virus** | **Genetic Group** | **Collection date** | **Reference viruses** | A/Cal | A/Bayern | A/Lviv | A/Chch | A/Astrak | A/St. P | A/St. P | A/HK | A/Sth Afr | A/Slov | A/Israel |
|  |  |  |  | 7/09 | 69/09 | N6/09 | 16/10 | 1/11 | 27/11 | 100/11 | 5659/12 | 3626/13 | 2903/2015 | Q-504/15 |
|  |  |  | **Passage history ^*^** | Egg | MDCK | MDCK | Egg | MDCK | Egg | Egg | MDCK | Egg | Egg | MDCK |
|  |  |  | **Ferret number** | F06/16 | F09/15 | F14/13 | F15/14 | F22/13 | F26/14 | F24/11 | F30/12 | F03/14 | F02/16 | F08/16 |
|  |  |  | **Genetic group** | **1** | **1** | **1** | **4** | **5** | **6** | **7** | **6A** | **6B** | **6B.1** | **6B.2** |
| **Reference viruses** |  |  |  |  |  |  |  |  |  |  |  |  |  |  |
| A/California/7/2009 Clone38-32 | 1 | 2009-04-09 | E3/E2 | **1280** | 640 | 640 | 640 | 1280 | 1280 | 5120 | 2560 | 2560 | 2560 | 2560 |
| A/Bayern/69/2009 (G155E) | 1 | 2009-07-01 | MDCK5/MDCK1 | 40 | **320** | 320 | 80 | 40 | 40 | 80 | 40 | 40 | 80 | < |
| A/Lviv/N6/2009 (G155E>G, D222G) | 1 | 2009-10-27 | MDCK4/SIAT1/MDCK3 | 160 | 1280 | **640** | 320 | 80 | 160 | 160 | 320 | 160 | 320 | 160 |
| A/Christchurch/16/2010 | 4 | 2010-07-12 | E1/E3 | 1280 | 1280 | 1280 | **2560** | 1280 | 640 | 2560 | 1280 | 2560 | 2560 | 2560 |
| A/Astrakhan/1/2011 | 5 | 2011-02-28 | MDCK1/MDCK5 | 1280 | 640 | 320 | 640 | **1280** | 1280 | 2560 | 2560 | 2560 | 2560 | 1280 |
| A/St. Petersburg/27/2011 | 6 | 2011-02-14 | E1/E3 | 1280 | 640 | 640 | 640 | 1280 | **1280** | 2560 | 2560 | 2560 | 2560 | 2560 |
| A/St. Petersburg/100/2011 | 7 | 2011-03-14 | E1/E3 | 1280 | 640 | 640 | 640 | 1280 | 1280 | **2560** | 2560 | 2560 | 1280 | 2560 |
| A/Hong Kong/5659/2012 | 6A | 2012-05-21 | MDCK4/MDCK2 | 320 | 160 | 160 | 160 | 320 | 320 | 1280 | **640** | 640 | 640 | 640 |
| A/South Africa/3626/2013 | 6B | 2013-06-06 | E1/E3 | 640 | 640 | 640 | 640 | 1280 | 1280 | 2560 | 1280 | **2560** | 1280 | 1280 |
| A/Slovenia/2903/2015 | 6B.1 | 2015-10-26 | E4 /E1 | 1280 | 640 | 640 | 640 | 1280 | 1280 | 5120 | 2560 | 2560 | **1280** | 2560 |
| A/Israel/Q-504/2015 | 6B.2 | 2015-12-15 | C1/MDCK2 | 1280 | 320 | 320 | 640 | 640 | 640 | 1280 | 1280 | 1280 | 2560 | **2560** |
| **CAR Test viruses** |  |  |  |  |  |  |  |  |  |  |  |  |  |  |
| A/Central African Republic/674/2015 | 6B | 2015-06-26 | MDCK1/MDCK2 | 1280 | 320 | 320 | 640 | 640 | 320 | 1280 | 1280 | 1280 | 1280 | 1280 |
| A/Central African Republic/809/2015 | 6B | 2015-08-11 | MDCK1/MDCK2 | 1280 | 320 | 320 | 640 | 640 | 320 | 1280 | 1280 | 1280 | 1280 | 1280 |
| A/Central African Republic/854/2015 | 6B | 2015-08-18 | MDCK1/MDCK1 | 640 | 320 | 640 | 320 | 640 | 320 | 1280 | 640 | 640 | 1280 | 1280 |
| A/Central African Republic/849/2015 | 6B | 2015-08-19 | MDCK1/MDCK1 | 1280 | 640 | 640 | 640 | 1280 | 640 | 2560 | 1280 | 1280 | 2560 | 1280 |
| A/Central African Republic/926/2015 | 6B | 2015-09-08 | MDCK1/MDCK1 | 640 | 320 | 320 | 320 | 640 | 320 | 1280 | 640 | 640 | 1280 | 1280 |
| A/Central African Republic/1282/2015 | 6B | 2015-11-26 | MDCK1/MDCK2 | 640 | 320 | 160 | 320 | 640 | 320 | 1280 | 1280 | 1280 | 2560 | 1280 |
| A/Central African Republic/851/2015 | 6B.1 | 2015-08-19 | MDCK1/MDCK3 | 640 | 320 | 160 | 320 | 640 | 640 | 1280 | 1280 | 1280 | 2560 | 1280 |

Vaccine virus is highlighted in yellow.

Homologous titres are shown in bold and underlined.

*E=Egg, MDCK= Madin Darby Canine Kidney cell-line, SIAT=MDCK-SIAT1 cell-line engineered to express increased levels of α-2,6-linked sialic acid receptors, C=mammalian cell of unspecified origin. The number of passages required to isolate a virus and produce sufficient HA titre to allow HI analyses is indicated behind each host/cell-line used.

< indicates a titre of <80.

**Table S3:** Antigenic characterization of post-2015 CAR A(H1N1)pdm09 isolates.

|  | |  |  |  | **Hemagglutination Inhibition Titre** | | | | | | | | | |
| --- | --- | --- | --- | --- | --- | --- | --- | --- | --- | --- | --- | --- | --- | --- |
|  | |  |  |  | **Post-infection ferret antisera** | | | | | | | | | |
| **Virus** |  | **Genetic group** | **Collection date** | **Reference viruses** | A/Mich 45/15 | A/Cal 7/09 | A/Bayern 69/09 | A/Lviv N6/09 | A/Astrak 1/11 | A/HK 5659/12 | A/Slov 2903/2015 | A/Paris 1447/17 | A/Swit 2656/17 | A/Swit 3330/17 |
|  |  |  |  | **Passage history ^*^** | Egg | Egg | MDCK | MDCK | MDCK | MDCK | Egg | MDCK | Egg | Egg |
|  |  |  |  | **Ferret number** | NIB F42/16 | F07/16 | F09/15 | F14/13 | F22/13 | F17/15 | F02/16 | F03/18 | F20/18 | F23/18 |
| **Reference viruses** | |  |  | **Genetic group** | **6B.1** | **1** | **1** | **1** | **5** | **6A** | **6B.1** | **6B.1** | **6B.1** | **6B.1** |
| A/Michigan/45/2015 | | 6B.1 | 2015-09-07 | E3/E3 | **640** | 1280 | 320 | 320 | 320 | 640 | 1280 | 2560 | 1280 | 640 |
| A/California/7/2009 | | 1 | 2009-04-09 | E3/E3 | 640 | **1280** | 320 | 320 | 320 | 640 | 640 | 1280 | 1280 | 320 |
| A/Bayern/69/2009 | | 1 | 2009-07-01 | MDCK5/MDCK1 | 40 | 80 | **320** | 320 | 40 | 40 | 80 | 320 | 160 | 80 |
| A/Lviv/N6/2009 | | 1 | 2009-10-27 | MDCK4/SIAT1/MDCK3 | 80 | 160 | 1280 | **1280** | 40 | 80 | 160 | 640 | 640 | 320 |
| A/Astrakhan/1/2011 | | 5 | 2011-02-28 | MDCK1/MDCK7 | 1280 | 1280 | 1280 | 640 | **1280** | 1280 | 2560 | 5120 | 2560 | 1280 |
| A/Hong Kong/5659/2012 | | 6A | 2012-05-21 | MDCK4/MDCK2 | 640 | 1280 | 320 | 160 | 320 | **640** | 1280 | 2560 | 1280 | 640 |
| A/Slovenia/2903/2015 | | 6B.1 | 2015-10-26 | E4/E2 | 640 | 1280 | 320 | 320 | 320 | 640 | **1280** | 1280 | 1280 | 640 |
| A/Paris/1447/2017 | | 6B.1 | 2017-10-20 | MDCK1/MDCK3 | 640 | 640 | 320 | 160 | 320 | 640 | 1280 | **2560** | 1280 | 640 |
| A/Switzerland/2656/2017 | | 6B.1 | 2017-12-21 | E5/E2 | 1280 | 1280 | 640 | 640 | 640 | 640 | 1280 | 2560 | **2560** | 1280 |
| A/Switzerland/3330/2017 | | 6B.1 | 2017-12-20 | E6/E1 | 640 | 640 | 320 | 320 | 320 | 640 | 1280 | 2560 | 1280 | **1280** |
| **CAR Test viruses** | |  |  |  |  |  |  |  |  |  |  |  |  |  |
| A/Central African Republic/618/2016 | | 6B.1 | 2016-06-02 | MDCK1/MDCK1 | 640 | 1280 | 320 | 320 | 640 | 320 | 640 | 2560 | ND | ND |
| A/Central African Republic/568/2018 | | 6B.1 | 2018-04-24 | MDCK1/MDCK2 | 1280 | 2560 | 640 | 320 | 640 | 1280 | 5120 | >5120 | >5120 | >5120 |
| A/Central African Republic/576/2018 | | n.s. | 2018-04-26 | MDCK1/MDCK2 | 640 | 1280 | 640 | 320 | 320 | 640 | 1280 | 2560 | 2560 | 1280 |
| A/Central African Republic/700/2018 | | n.s. | 2018-05-24 | MDCK1/MDCK2 | 640 | 640 | 320 | 160 | 320 | 320 | 640 | 1280 | 1280 | 640 |
| A/Central African Republic/804/2018 | | 6B.1 | 2018-06-18 | MDCK1/MDCK1 | 1280 | 2560 | 640 | 320 | 640 | 1280 | 2560 | 5120 | 2560 | 1280 |
| A/Central African Republic/843/2018 | | 6B.1 | 2018-06-29 | MDCK1/MDCK1 | 640 | 1280 | 320 | 320 | 320 | 640 | 1280 | 2560 | 2560 | 1280 |

Vaccine virus is highlighted in yellow.

Homologous titres are shown in bold and underlined.

*E=Egg, MDCK= Madin Darby Canine Kidney cell-line, SIAT=MDCK-SIAT1 cell-line engineered to express increased levels of α-2,6-linked sialic acid receptors. The number of passages required to isolate a virus and produce sufficient HA titre to allow HI analyses is indicated behind each host/cell-line used.

n.s. These two viruses were not sequenced so a genetic group could not be assigned.

ND=Not Done

**Table S4**: Susceptibility of CAR type A influenza viruses to neuraminidase inhibitors.

| **Virus** | **Collection date (dd/mm/yyyy)** | **A/subtype** | **Oseltamivir** | | **Zanamivir** | | **Reduced susceptibility marker^!^** |
| --- | --- | --- | --- | --- | --- | --- | --- |
|  |  |  | **IC_50_*** | **Sensitivity^$^** | **IC_50_*** | **Sensitivity^$^** |  |
| A/Central African Republic/674/2015 | 26/06/2015 | H1pdm09 | 1.75 | NI | 0.65 | NI | None |
| A/Central African Republic/809/2015 | 11/08/2015 | H1pdm09 | 0.98 | NI | 0.44 | NI | None |
| A/Central African Republic/854/2015 | 18/08/2015 | H1pdm09 | 1.35 | NI | 0.52 | NI | None |
| A/Central African Republic/849/2015 | 19/08/2015 | H1pdm09 | 1.54 | NI | 0.61 | NI | None |
| A/Central African Republic/851/2015 | 19/08/2015 | H1pdm09 | 2.52 | NI | 1.26 | NI | None |
| A/Central African Republic/926/2015 | 08/09/2015 | H1pdm09 | 1.34 | NI | 0.50 | NI | None |
| A/Central African Republic/1282/2015 | 26/11/2015 | H1pdm09 | 1.25 | NI | 0.49 | NI | None |
| A/Central African Republic/618/2016 | 02/06/2016 | H1pdm09 | 820.59 | HRI | 2.23 | NI | H275Y |
| A/Central African Republic/737/2016 | 04/07/2016 | H1pdm09 | Virus not recovered (cs sequence) | | | | H275Y |
| A/Central African Republic/568/2018 | 24/04/2018 | H1pdm09 | Insufficient NA activity | | | | None |
| A/Central African Republic/570/2018 | 25/04/2018 | H1pdm09 | Virus not recovered (cs sequence) | | | | None |
| A/Central African Republic/576/2018 | 26/04/2018 | H1pdm09 | 1.57 | NI | 0.5 | NI | None |
| A/Central African Republic/700/2018 | 24/05/2018 | H1pdm09 | 1.17 | NI | 0.57 | NI | None |
| A/Central African Republic/804/2018 | 18/06/2018 | H1pdm09 | 1.00 | NI | 0.29 | NI | None |
| A/Central African Republic/843/2018 | 29/06/2018 | H1pdm09 | 1.66 | NI | 0.50 | NI | None |
|  |  |  |  |  |  |  |  |
| A/Central African Republic/878/2015 | 25/08/2015 | H3 | 0.26 | NI | 0.35 | NI | None |
| A/Central African Republic/808/2016 | 22/07/2016 | H3 | 0.22 | NI | 0.38 | NI | None |
| A/Central African Republic/968/2016 | 08/08/2016 | H3 | 0.37 | NI | 0.33 | NI | None |
| A/Central African Republic/972/2016 | 10/08/2016 | H3 | Virus not recovered (cs sequence) | | | | None |

* IC_50_: Inhibitory concentration of antiviral that reduces 50% of the NA activity of the virus.

^$^ Phenotype: NI (normal inhibition), RI (reduced inhibition), HRI (highly reduced inhibition) as defined in WHO Wkly. Epidemiol. Rec. (2012) 87, 369-374

^!^ Listing of NA amino acid substitutions associated with reduced inhibition by neuraminidase inhibitors (reduced susceptibility markers) for seasonal influenza viruses can be found on the WHO website:

<https://www.who.int/teams/global-influenza-programme/laboratory-network/quality-assurance/antiviral-susceptibility-influenza/neuraminidase-inhibitor>
